# Supplementary material for: Clustering rare diseases within an ontology-enriched knowledge graph
Source: bioRxiv. 2023 Feb 16:2023.02.15.528673. Preprint. [Version 1] doi: 10.1101/2023.02.15.528673 (PMC9949046; doi:10.1101/2023.02.15.528673)
Supplement: Supplement 1 [file NIHPP2023.02.15.528673v1-supplement-1.pdf]

bioRxiv preprint doi: https://doi.org/10.1101/2023.02.15.528673; this version posted February 16, 2023. The copyright holder for this preprint (which was not certified by peer review) is the author/funder, who has granted bioRxiv a license to display the preprint in perpetuity. It is made available under aCC-BY 4.0 International license.

Supplemental Figures

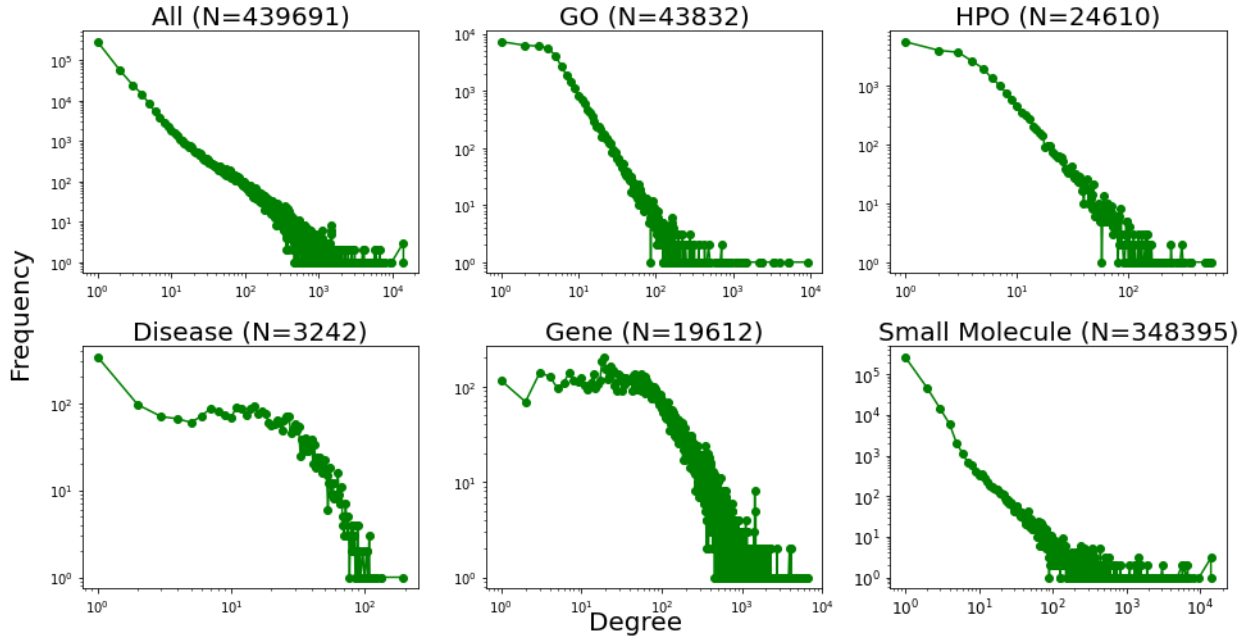

Figure S1 The degree distribution of various node types within the rare disease knowledge graph. Both y and x axes show the raw data values on a log-scale.

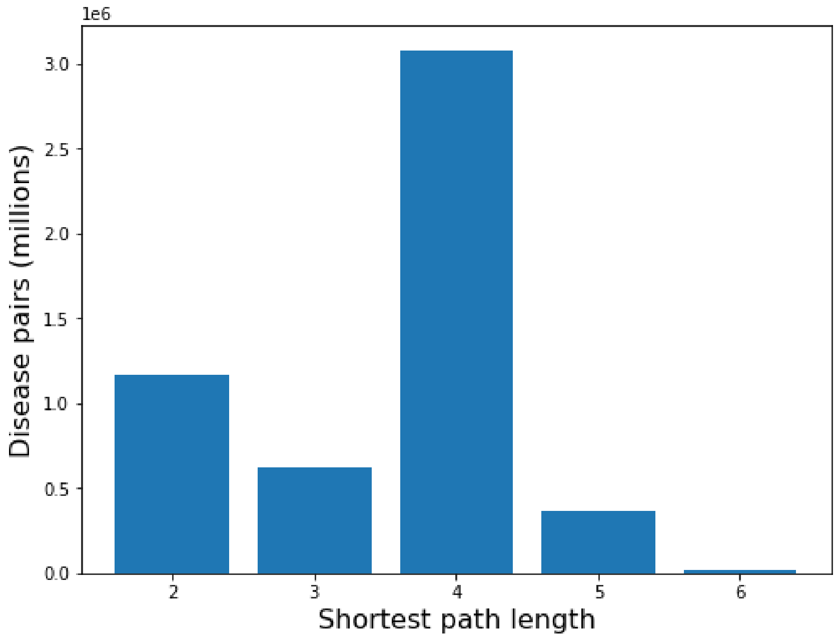

Figure S2 The distribution of shortest path lengths between all disease pairs within the rare disease knowledge graph

bioRxiv preprint doi: https://doi.org/10.1101/2023.02.15.528673; this version posted February 16, 2023. The copyright holder for this preprint (which was not certified by peer review) is the author/funder, who has granted bioRxiv a license to display the preprint in perpetuity. It is made available under aCC-BY 4.0 International license.

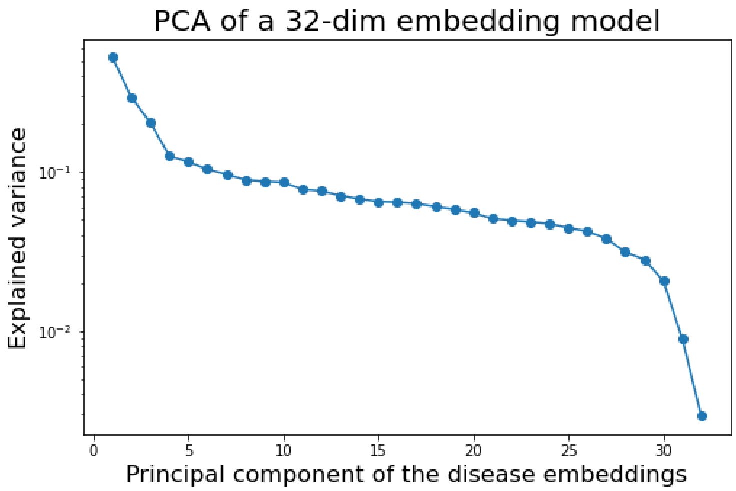

Figure S3 The variance in embedding values explained by successive principal component of the disease embedding matrix for an embedding dimension of 32.

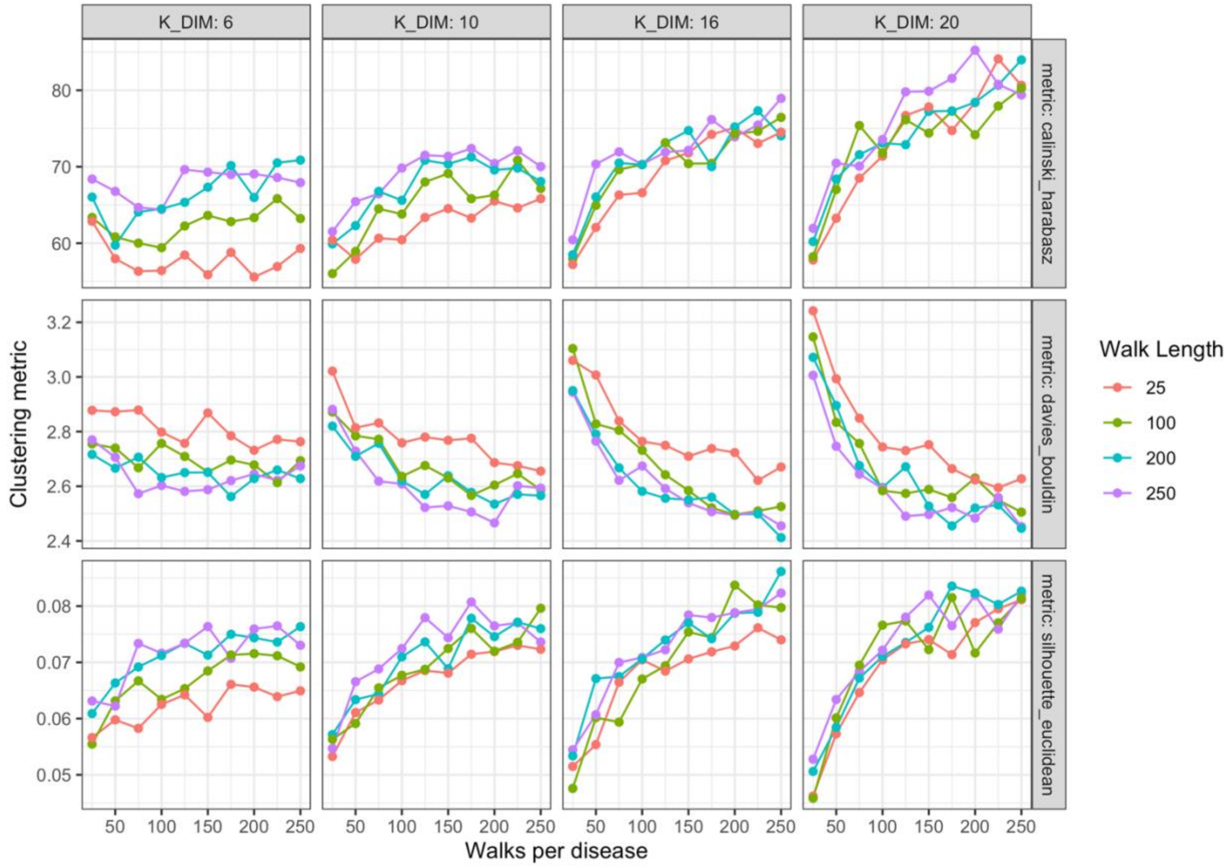

Figure S4: Internal clustering metrics as function of node embedding model hyper parameters. The colors of each line signify the length of the random walks used to construct the disease corpus. The chart columns vary the embedding context window (K_DIM). The chart rows show the three different clustering metric used, which are each plotted over the number of random walks generated per disease.

bioRxiv preprint doi: https://doi.org/10.1101/2023.02.15.528673; this version posted February 16, 2023. The copyright holder for this preprint (which was not certified by peer review) is the author/funder, who has granted bioRxiv a license to display the preprint in perpetuity. It is made available under aCC-BY 4.0 International license.

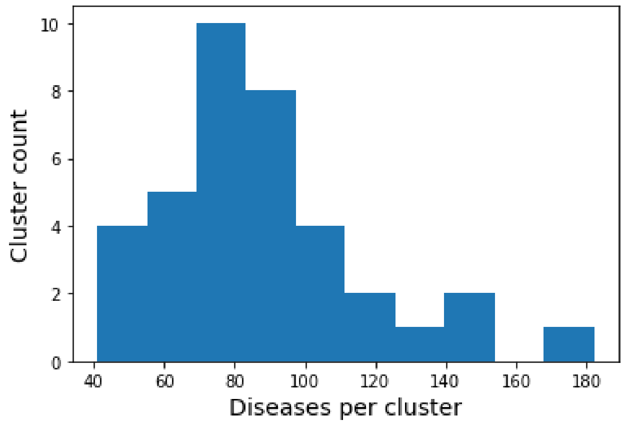

Figure S5 A histogram displaying the distribution of the number of diseases in each of the final clusters
